# Understanding barriers for research involvement among paediatric trainees: a mixed methods study

**DOI:** 10.1186/s12909-018-1263-6

**Published:** 2018-07-13

**Authors:** Khurram Mustafa, Carolyn Czoski Murray, Emma Nicklin, Adam Glaser, Jacqueline Andrews

**Affiliations:** 1 Leeds Teaching Hospitals NHS Trust, Leeds, UK; 2 Leeds Institute of Health Sciences, Leeds, UK; 3 Leeds Institute of Cancer and Pathology, Leeds, UK; 4 University of Leeds, Leeds Institute of Cancer and Pathology,, Leeds, UK

**Keywords:** Barriers to research, Paediatrics, Training scheme/pathway, Research culture, Support, Training, Paediatric research, Child health research

## Abstract

**Background:**

Child Health research is reported to be at worryingly low level by the Royal College of Paediatrics and Child Health. Recent survey showed that 54.5% of paediatric consultants in the United Kingdom do not do any research at all. We conducted a mixed methods study to understand barriers and facilitators for research involvement among paediatric trainees who are going to fill these consultant posts in the future.

**Methods:**

A questionnaire based on a validated index for research and development was completed by 136 paediatric trainees within a region in the North of England (Yorkshire and Humber). Twelve semi-structured interviews were conducted with stratified purposive sampling. Descriptive statistics and Chi-Square test for independence were used for quantitative analysis. Thematic content analysis was done for interviews based on analysis method framework.

**Results:**

136 out of 396 trainees responded to the survey. There was a significant relationship between confidence in using research in practice and ability to understand research terminology. This was not related to research experience or training. Males were significantly more likely to have presented a research paper, know how research influences practice and have more confidence in using research in practice than females. There was no significant relationship between gender and research training or highest qualification. Time constraints and lack of academic culture were the most frequently mentioned barriers in the survey.

Over-arching themes identified from the interviews were related to lack of academic culture, opportunities provided in current training scheme and constraints related to time availability along with workforce management.

**Conclusion:**

Paediatric research requires a supportive academic culture with more flexibility in training scheme and immediate attention to a pressing staffing crisis.

**Electronic supplementary material:**

The online version of this article (10.1186/s12909-018-1263-6) contains supplementary material, which is available to authorized users.

## Background

Child Health research has been reported to be at worryingly low level by the Vice president of Royal College of Paediatrics and Child Health, United Kingdom [1]. The United Kingdom has been at the forefront of research in child health and placing evidence at the centre stage for clinical practice [2–4]. Between 2000 and 2011, the proportion of academic paediatricians in the UK has fallen from 11.3 to 5.9% of the consultant workforce [5].

Around 82% of paediatric consultants in the National Health Service do not have research as part of their programmed activities and 54% do not do any research at all [1]. If this trend continues, we will be deprived of strong leadership in paediatric research in the near future, as 50% of current researchers are expected to retire in the next 10 years [6]. Internationally, the number of adult trials published annually in high-impact general medical journals has doubled during the past 20 years, with virtually no change in paediatric trials [7].

Although, there has been a survey to estimate research involvement among consultants, there are no studies investigating the trend among paediatric trainees in the UK who will be in positions to lead the specialty in coming years. We conducted a mixed methods study to understand barriers and facilitators for research involvement among paediatric trainees in a specialist training regional group in the North of England, Yorkshire and the Humber deanery, which is one of the largest training boards within Health Education England [8].

We chose to undertake a survey and qualitative interviews to enable us to gain more insights into the question than would be available from using one approach [9]. We have referred to the results from each of the studies to give additional context to the results.

## Methods

### Survey

**Fig. 1:**
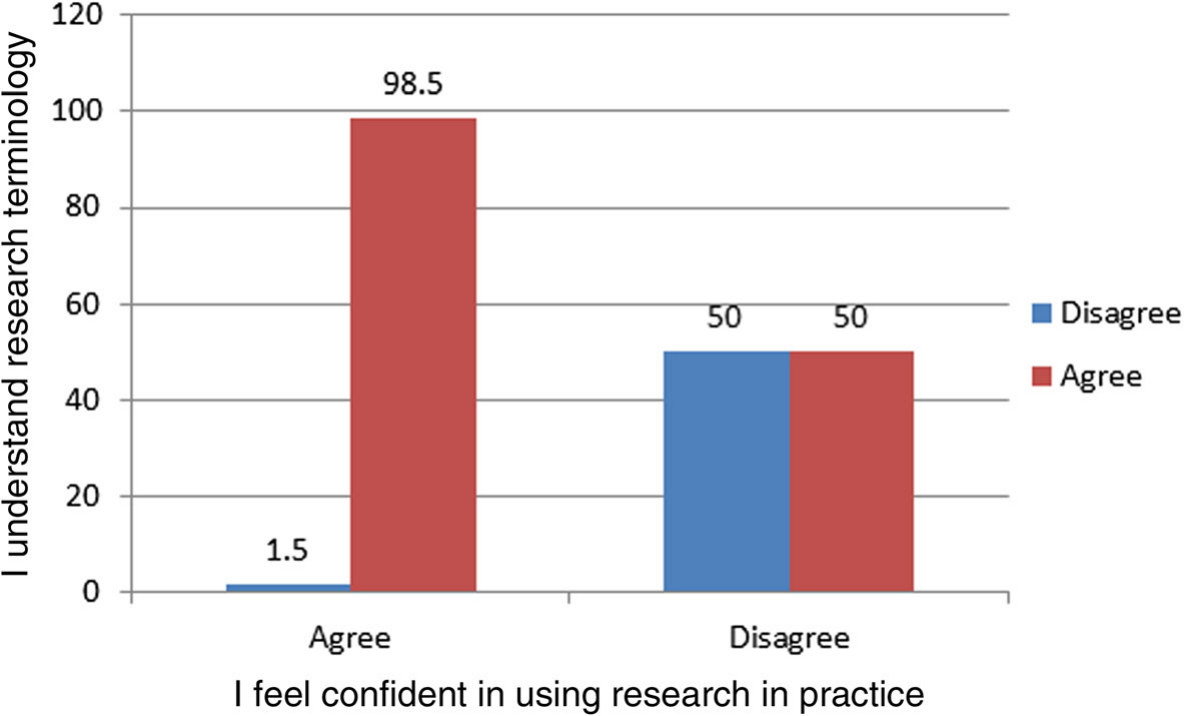
Relationship between confidence in using research and understanding terminology. 98.5% respondents who understood research terminology also felt confident about using research in practice

A survey questionnaire, based on a validated index for research and development [10] was disseminated between December 2016 and April 2017 to all paediatric trainees within Yorkshire and Humber (Fig. 1). The questionnaire covered demographics including gender, years of experience, highest qualification, training and any research experience. Participants were asked about research and development culture in the workplace including their own confidence in applying research in practice. We included descriptive free text questions to allow for additional information about potential barriers and facilitators to involvement in research.

An online link for the questionnaire on Bristol Online Survey (BOS) [11] was emailed to trainees via the training board and a reminder email was also sent. Introductory presentations were given at regional teaching days where paper based questionnaires were also disseminated and completed by those who had not participated online. This was then transferred to the online survey.

### Statistical analysis

Data were exported from BOS and analysed in SPSS version 24.0 [12]. Descriptive statistics were used to assess participants’ characteristics, research background/experience and their feelings toward research. To analyse the differences between categorical variables (i.e. gender) the Chi-Square Test for Independence was used. Significance levels were set at *p* < 0.05 for all comparisons.

### Semi-structured interviews

A topic guide was designed, based on data from existing literature, especially recent workforce survey conducted by the RCPCH and validated index for research and development culture (Additional file 1: Appendix 1). Two pilot interviews were carried out with paediatric trainees fitting the sampling framework, to identify any areas for improvement in the initial topic guide. The responses from these interviews were also included in the analysis.

We developed a sampling framework to ensure representation across gender, stage of training and research experience. We planned to undertake 15 interviews to allow for expression of a range of views. Participants were recruited using stratified purposive sampling methods [13].

Potential participants were given an information leaflet before obtaining written consent (Additional file 2: Appendix 2). Interviews lasted for 29 min on average, were electronically recorded and then transcribed verbatim by the interviewer (KM). All transcripts were compared to audio recordings for confirmation of accuracy. A second researcher, (CCM), listened to a sample of recordings and compared the transcripts. 12 participants were interviewed by one researcher (KM) and further interviews were discontinued with consensus between authors that data saturation was achieved with no new emerging themes.

Thematic content analysis was undertaken to classify data into main themes with consensus between two reviewers. Data was initially organized cross-sectionally and synthesized as categories were refined based on emerging themes, following the analysis method framework [14]. Familiarization with data was achieved [15] by both reviewers (KM, CCM) through involvement in literature review, designing topic guide and performing interim analysis after pilot interviews. KM conducted all interviews and is a level 2 paediatric trainee, which allowed further insight into accounts and language used by participants.

A thematic framework was synthesized and data was indexed [16] into main themes and subthemes based on descriptive and explanatory accounts. Evolving themes were refined through constantly revisiting original data and including new concepts and categories. Once the charted data was investigated, patterns and associations were identified based on recurrence of themes and links between sets of phenomenon and perspectives, as well as any relation with pre-defined typologies including clinical and academic experience.

## Results

### Survey

The survey questionnaire was filled by 136 out of 396 trainees (80 online and 56 on paper) with a response rate of 34.3%. Gender distribution was similar to the target population with more female trainees. Respondents’ demographics are shown in Table 1.

**Table 1:** Demographics

Gender:	
Male: 37 = 27.2%	
Female: 99 = 72.8%	
Clinical experience in years	Highest qualifications
< 5 years: 51 = 37.5%	Phd: 1 = 0.7%
5–10 years: 73 = 53.7%	Masters: 16 = 11.8%
> 10 years: 11 = 8.1%	MD: 8 = 5.9%
Unavailable: 1 = 0.7%	PGCert: 11 = 8.1%
	PGDip: 20 = 14.7%
	Degree: 78 = 57.4%
	Other: 2 = 1.5%

One respondent had completed doctorate degree while 41.2% reported having additional qualification to a medical degree including bachelors, masters or post graduate diplomas (membership exams were not included as additional qualifications). The average number of years of clinical experience among respondents was 6.9 +/− 2.836. There was no significant relationship between participant’s confidence in using research in their practice and their highest qualification or years of clinical experience.

Research terminology was not well understood by 26.5% of participants. The relationship between respondents feeling confident about using research in practice and whether they felt they understood research terminology was statistically significant, *p* < 0.001. (Fig. 1:

Relationship between confidence in using research and understanding terminology shows that 98.5% respondents who understood research terminology also felt confident about using research in practice) This did not appear to be linked to research experience or training.

There was a statistically significant relationship between gender and respondents who had experience of presenting a conference paper (*p* = 0.001). Males were more likely to have presented a research paper (43.2%) in comparison to females (16.2%). Almost half (48.5%) of respondents did not have any experience of research including presentations, publications nor grant applications. No relationship was found between gender and the frequency of respondents that had been an author of a peer reviewed journal (*p* = 0.43).

**Fig. 2:**
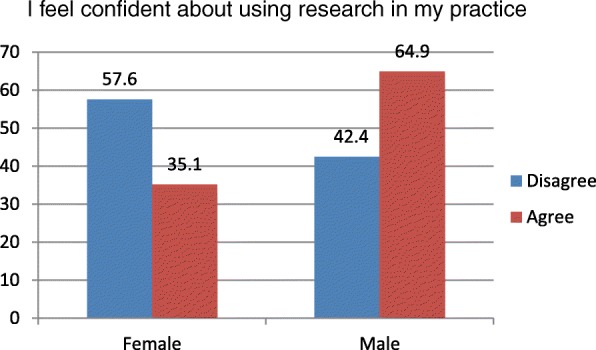
Title: Gender difference in confidence about using research in practice. Females felt less confident than male respondents about using research in practice

A significant relationship between gender and respondents knowledge of how research influenced practice existed (*p*= 0.03). Females (57.6%) also felt less confident than males (35.1%) about using research in their practice (Fig. 2: Gender difference in confidence about using research in practice). There was no statistically significant relationship between gender, and working hours or research training and highest qualification.

Respondents had the opportunity to enter free text responses about perceived barriers and facilitators for research involvement. We invited suggestions for what kind of training would be most beneficial. These responses were categorized into major themes based on frequency (Table 2).

**Table 2:** Themes from survey questionnaire

	Theme	*N*	%
Facilitators	Culture and support	71	52.2%
	Time	31	22.8%
	Opportunities and Exposure	15	11.0%
	Research training provided at PGD and STEPP	12	8.8%
Barriers	Time, rota issues and clinical commitments	117	86.0%
	Culture, support	41	30.1%
	Accessibility and availability of opportunities	25	18.3%
	Lack of flexibility in training and other assessments	11	8.1%

Time limitation along with rota issues and clinical commitments was mentioned by 86.0% of respondents as a barrier. The second most frequently reported barrier, by 30.1% of respondents, was a lack of academic culture. Respondents, 52.2%, reported that a more positive and supportive academic culture would facilitate their involvement in research, while 22.8% suggested that protected or allocated time for research would be helpful. Respondents, 46.3%, also wanted more training in research skills, including how to initiate a research project. Opportunities to get involved and exposure to practical aspects of research could make a real difference, according to 22.8% of respondents.

### Interviews

Three over-arching themes for barriers in research involvement identified by the two researchers (KM,CCM), were associated with academic culture, opportunities in current training scheme along with constraints related to time and workforce management. (Table 3) Suggestions to overcome these barriers emerged under these categories.

**Table 3:** Themes and subthemes from interviews

Theme	Subtheme	
1. Academic culture	1.1 Mentorship / leadership	
	1.2 Role modelling	
	1.3 Active exclusion	
	1.4 Academic pathway	
	1.5 Perception of a gap or conflict between research and clinical paediatrics	
	1. 6 Support and recognition	
	1.7 Perceptions	1.7a Unattainability
		1.7b Certain personalities
		1.7c Boring / dry
2. Training scheme and opportunities	2.1 Impact of run-through	
	2.2 Other competencies (Tick box exercises)	
	2.3 Lack of flexibility	
	2.4 Availability of opportunities	
	2.5 Advertisement of opportunities	
	2.6 Inequality / Inequity	
	2.7 Impression of training board	
3. Constraints	3.1 Time	
	3.2 Short staffing	

There were no major differences in perceptions between participants with different levels of clinical or research experience. Certain subthemes were discussed more in-depth by those with higher academic experience. Further interviews were not conducted with consensus between the researchers when no new information was identified, which we defined as data saturation.

### Culture

The overarching theme identified as a major factor contributing in trainees’ involvement in research was influence of an academic culture.

“I think part of it is the culture, it’s (paediatrics) not a traditionally research driven specialty.” (Interviewee 4)

A difference in research participation and promotion was noted between different hospitals and departments with strong leadership in certain units driving the academic culture. All interviewees, regardless of their previous experience in research perceived a lack of interest and visibility of research in general paediatrics. One trainee had only attended two journal club sessions in 5 years of paediatric training.

“you don’t really see it (research) happening first hand so you kind of hear about it and you know it’s going on but not really seeing it.” (Interviewee 1).

Participants believed that this needs to be addressed in a top to bottom approach because the lack of interest among juniors is driven from a lack of motivation among consultants thereby forming a vicious cycle. All trainees who had participated in research, mentioned at-least one senior or consultant who had been crucial in their academic progress, sometimes from the very beginning of training in medical schools.

On the other hand, participants who had not actively participated in research were unsure about how to pursue their interest. A few trainees mentioned, how despite taking interest in research, they were actively excluded from participation.

“but as an SHO you weren’t included in that (research) at all. I remember being specifically excluded from helping with the studies” (Interviewee 3).

All interviewees perceived that there is a division between clinicians and researchers with a lack of understanding of academia in the clinical environment giving rise to misconceptions and myths about research.

“I think there is always a like split between clinicians and researchers, researchers look down on clinicians and clinicians look down a bit on academics.” (Interviewee 5).

“You (researchers) are definitely seen as a hindrance (by ward staff), you feel you are in the way of the care of the child.” (Interviewee 8).

Some subthemes were discussed from different perspectives by those with and without active involvement in research including impact of academic involvement on clinical performance. Some trainees who had not been actively involved in research believed that it is difficult to be a sound clinician and an active researcher simultaneously. There were concerns related to understanding of incorporation of research into clinical practice. Others, however, said that research has impacted positively on their clinical work, helping to understand the theory behind clinical practice.

“It (research) impacted on my clinical work because I was able to look at the knowledge…. I felt like I could really impact clinically.” (Interviewee 7).

Participants, who had undertaken research as part of a higher degree, recurrently suggested that they felt unsupported in clinical environments, during and after completing their research and were required to make sacrifices along the way without any support or appreciation.

“The reality is that I haven’t burnt out but I feel quite depressed about the situation because I feel as you can see, I’ve put in a lot of work and I’ve sacrificed a lot to get where I got.” (Interviewee 8).

There was a realization that clinical commitments should take priority, but even being able to use their free-time for research was challenging due to external pressures.

“It’s even just being allowed to spend my spare time to do research brought me to tears a couple of times by people saying no you can’t do that, why not? I am allowed to locum, I am allowed to pick up extra shifts to cover the unit but I am not allowed to pick up extra work to develop my skills in research.” (Interviewee 3).

Due to the lack of exposure and previous involvement, interviewees reported that research is perceived as dry or uninteresting. There was a general perception that researchers have a specific personality and are more intelligent, focused and determined as well as willing to sacrifice their personal and social lives.

“One or two special people that are really intelligent and have really a lot of passion for research they are involved but the rest of us like mere mortals kind of don’t get involved with it.” (Interviewee 10).

One of the most recurring recommendations to improve research culture was to provide visible leadership, mentorship and role-models that participants could identify with.

### Current training scheme and available opportunities

There was a general perception that the current training scheme provides little motivation and stimulation for ‘extracurricular’ activities and there is very limited incentive in building a profile outside clinical competencies. Interest in certain subspecialties was noted to be a driver for academic involvement with the perception of a strong academic culture and an expectation for trainees to have research experience. Run-through training, which means that paediatric trainees can complete their training from ST1 (1st year after foundation) to ST8 (8th year) without any need for re-application, was recognized as beneficial in providing stability with personal lives. It was regarded as making trainees laid back, as they could follow a ‘conveyer belt’ without having much competition or requirement to do something ‘extra’.

“I think because we are run-through it’s very easy to not do it (research). So therefore people choose for not doing it, so it’s not really there. It’s part of a culture that’s gone forwards that actually you don’t need to, so why would you and for those who don’t have active interest, it’s just been forgotten… I don’t think we have any drive necessarily to develop our CV.” (Interviewee 12).

Another limitation was a lack of flexibility in paediatric training. Trainees mentioned the benefits of having pre-allocated study leaves and standardized teaching. It was considered by some participants, to limit their chances for pursuing individual interests.

Academic competency was not considered to be a priority in training. Some of the required competencies, including audits, were termed as ‘tick-box exercises’ with little benefit seen in their influence on professional development.

“I think people have been keen for us to do audit but I have to admit I don’t think they are that bothered about seeing it through, I think you should just be seen to do something…Yeah and I think it’s probably the same everywhere, you should do something but it doesn’t really matter what it is.” (Interviewee 12).

Trainees shared the opinion that there were very few opportunities to get involved in research and even those available were not properly advertised. On many occasions, suitable trainees were disadvantaged due to lack of networking or awareness of opportunities. This idea was more widely shared between those who were actively involved in research along with a frustration with lack of opportunities to progress further in academia in the region.

“I don’t know if I am cynical but it appears to be that the favourable local candidate gets the job.” (Interviewee 8).

When asked about comparison with other areas, all interviewees seemed to think that London, Oxford and Cambridge training regions potentially have more supervisors, projects and access to funding for research. The lack of research in paediatrics was evaluated by an interviewee in comparison with other specialties.

“I went to the (name) academic event, there are not many paediatricians, most people are (from) other specialties, I did feel stupid. I feel as paediatricians you don’t do like everybody else, proper projects or proper lab, they have supervisors on this, they have got grant for that, we have nothing like that in paediatrics” (Interviewee 12).

Trainees had mixed ideas about how they believe research is supported by the local training board. They appreciated the pressures of service provision and that the programme directors have to meet requirements from different hospitals, but there was a general impression that research is not valued as a priority.

“I guess they don’t pride themselves on it whereas friends that are sort of Oxbridge - London triangle; it’s very much like why haven’t you got a paper, of course you need to do some research, of course, it’s an important thing to do. Whereas in this Deanery, it’s less pushed by the powers.” (Interviewee 4).

Participants recommended that research training should be more consistent with recognition of individual interests and provision of more specific opportunities. There is a need for more support and encouragement from training providers for those who are interested, actively involved or wish to continue their participation in research.

“I don’t think the programme supports people to do research I don’t think it encourages people to learn about research.” (Interviewee 3).

### Constraints

Time constraint due to clinical and portfolio commitments was one of the main barriers to trainees’ involvement in research. Those actively involved in research appreciated that academic commitments had to be met at the expense of their own time and seemed to have accepted this as a reality. Although, they acknowledged that it is very hard and resulted in sacrifices to their personal lives.

“To continue (research) means that there is a sacrifice to your own personal time which means a sacrifice to your personal life and I think … it was a big sacrifice to my personal life, so I think anyone could do it but you have to be willing to make a sacrifice along the way.” (Interviewee 8).

All interviewees discussed pressures due to current short staffing in paediatrics and recognized that immediate measures are required to deal with this. Trainees also appreciated that current pressures due to uncovered rotas further limit their availability and opportunities to get involved in research. Researchers shared how they had been asked to provide clinical care during designated academic time.

“Anyone can suggest research to us but actually to have an awareness that it does take time and give us the opportunities or some time in which to do it, that would make a difference.” (Interviewee 10).

## Discussion

This study describes the current academic atmosphere within paediatrics in a large region within the NHS and barriers for research involvement perceived by future leaders in this specialty. An incorporation of mixed methods allowed for representation of a wide population with deeper understanding of perceptions among participants.

We demonstrated similar emerging themes through both quantitative and qualitative methods. One of the most commonly identified barriers was time constraint further affected by short staffing in paediatrics. A need for stronger academic culture in paediatrics was identified by all interviewees and most frequently perceived as a major facilitator by survey respondents. These findings are in keeping with previous studies in paediatric clinicians and emergency medicine doctors outside the UK [17, 18].

The Royal College of Physicians conducted a survey in 2015 which found that at least 46% of consultants had assisted in a clinical study [19] in comparison to paediatrics where half of the consultants don’t do research at all. It is interesting to note that in another specialty in Canada where research participation is mandated, the majority of trainees agreed that they would rather prefer to participate in a different educational programme [20]. Therefore a good balance of academic culture that promotes research but also recognizes individual interests and career aspirations will be most fruitful in supporting those with active interest in research and providing them with an encouraging environment to flourish in. There is a need to ensure that trainees actively involved in research are well supported and feel valued both in academic and clinical environments along with provision of more academic posts [21].

One interesting finding from the survey was lower confidence in utilizing research in practice among female respondents compared to male trainees. There is a considerable body of literature which addresses these issues and there remains considerable debate on how to bring about change. Recent work by colleagues in Leeds as part of the Athena Swan initiative highlights that the solutions proposed may also contribute to women feeling less confident in their skills [22]. They suggest that ‘Family friendly’ policies on the whole do not challenge attitudes and may contribute to women further internalising the message that they are not expected to be as competent as their male colleagues. The language of higher education and research particularly in the emphasis on ‘excellence’ is not gender neutral and tends to be more accessible to males [23]. ^.^We hope that our study will provide further ground for future research in this subject.

Paediatric training in the UK is provided as a run-through programme over 8 years following a 2 years foundation programme after graduating from medical school. Once recruited into the scheme, trainees can acquire a certificate of completion after 8 years without any reapplication process. Some might apply for nationally competitive posts in subspecialties, usually at year 5 or 6 of training. There were concerns that this model doesn’t motivate trainees for improvement in their portfolios, unless they wish to apply for certain subspecialties. Academic experience is not considered a requirement to apply for most for these specialties.

The Paediatric STEPP is a regional based education programme for level 1 trainees (ST1 to ST3), hosted across many centres within Yorkshire and Humber. These are structured education sessions delivered by selected speakers and experts which include 4 days on introduction to evidence based medicine over 3 years [24]. At level 2, (ST4, ST5) all trainees have to attend a post graduate diploma in child health which is a unique opportunity offered in this region. A module for research methods and one for evidence based medicine is offered within the diploma curriculum. Most interviewees believed that the current structure of standardized training in the region is helpful for research involvement, although there should be recognition of individual needs, especially for those with existing academic experience and competency.

The latest workforce survey done by the RCPCH [1] suggests that almost a quarter of paediatric posts were vacant at senior trainee level. This stretches the existing training cohort with very limited time, if any, left for anything outside clinical commitments. As pointed out by participants in this study, there is a need for drastic measures to address the situation in order to provide time and staffing to support researchers in paediatrics.

### Limitations of study and future research

We recognize that this study is limited due to a relatively low response rate for the survey. This is not dissimilar to previous studies exploring similar topics [20]. It indirectly suggests a lack of culture of participation and encouragement for research activity among paediatric trainees. This study was based in one of the largest training boards in the UK and there are no compelling reasons for it not to be reflective of a wider population. We were not identifying new information in our interviews; potentially if we had continued in a wider sample in a different geographical area, we may have identified additional themes. There is a perception that these findings may differ in some areas like London, with a more visible research culture. A larger national or multi-regional study is needed to further evaluate the barriers and facilitators for research involvement among paediatric trainees.

## Conclusion

NHS England prioritises the creation of an environment that fosters research and innovation [25]. In 2012, the RCPCH published recommendations to improve child health research that included the need for more collaborative efforts and the building of greater research capacity. The importance of provision of training, to education and guidance junior doctors was emphasized [5]. Concerns raised by trainees in this study about the current training structure, workforce management, lack of appreciation and support for trainees involved in research all suggest these recommendations may not be being implemented. The findings would suggest that paediatric research in the NHS requires urgent attention to develop a supportive academic culture with more flexibility in training scheme supported by immediate attention to the current staffing crisis.

## Additional files

Additional file 1: **Appendix 1.** Information Leaflet. Information provided to participants before written consent. (DOCX 18 kb)

Additional file 2: **Appendix 2.** Topic guide. Topic guide used for semi-structured one to one interviews. (DOCX 19 kb)

## Abbreviations

BOS: Bristol Online Survey
CV: Curriculum Vitae
NHS: National Health Service
PGCert: Post Graduate Certificate
PGDip: Post Graduate Diploma
RCPCH: Royal College of Paediatrics and Child Health.
ST: Specialty Trainee
STEPP: Specialty Trainee Education Programme in paediatrics
