# Supplementary material for: Understanding barriers for research involvement among paediatric trainees: a mixed methods study
Source: BMC Med Educ. 2018 Jul 13;18:165. doi: 10.1186/s12909-018-1263-6 (PMC6044020; doi:10.1186/s12909-018-1263-6)
Supplement: Supplementary file 1 — Appendix 1. Information Leaflet. Information provided to participants before written consent. (DOCX 18 kb) [file 12909_2018_1263_MOESM1_ESM.docx]

**BROAD: B**arriers in **R**esearch f**O**r p**A**edatric **D**octors in training

TOPIC GUIDE: O ne to one interview

**Welcome**

Aim of Interview: To gain an understanding of the barriers and facilitators in research as perceived by paediatric trainees in Yorkshire and the Humber.

Ground rules: confidentiality, consent, request to audio record the interview.

**Questions**

1. What is your current level / stage of training?
2. Specialty and hospital currently working in?
3. Can you talk to me about your clinical experience so far? Where did you graduate from and where have you worked so far?
4. Have you worked outside Yorkshire and The Humber as a doctor?
5. Have you got any additional qualifications to MBBS?
6. What is your aspiration or career plan as a paediatric doctor?
7. Have you ever been involved in research?
8. How much was your involvement?
9. How is/ was your experience?
10. Themes to talk about:
    1. **Skills:** How confident are you with your understanding of research?
    2. **Training?** What training have you received in research? How satisfied are you with the training? Has it been helpful?
    3. **Support:** Research friendly Environment? Have you worked in an environment that encourages undertaking research?
    4. **Opportunities?** What opportunities have been made available to you? Do you think they are enough? Is there a difference in opportunities in the region compared to other areas?
    5. **Challenges:** Time ? staffing? etc
11. If planning to get involved in research, Have you thought about how are you going to approach it?
12. Do you think there are any barriers in pursuing your plan? What are those?
13. In your opinion, what works best? What are the barriers?
14. Have you got any suggestions for how to improve involvement of paediatric trainees in research?
15. How can the deanery / RCPCH best support you?
