# Supplementary material for: Understanding barriers for research involvement among paediatric trainees: a mixed methods study
Source: BMC Med Educ. 2018 Jul 13;18:165. doi: 10.1186/s12909-018-1263-6 (PMC6044020; doi:10.1186/s12909-018-1263-6)
Supplement: Supplementary file 2 — Appendix 2. Topic guide. Topic guide used for semi-structured one to one interviews. (DOCX 19 kb) [file 12909_2018_1263_MOESM2_ESM.docx]

**Participant Information Sheet**

**Study title: BROAD: B**arriers in **R**esearch f**O**r p**A**edatric **D**octors in training

**Invitation:**

You are invited to take part in a study aimed at understanding the barriers in research for paediatric trainees.

Before you decide whether or not you want to be involved, it is important for you to understand why the study is being done and what it will involve. We would be grateful if you would take the time to read this information sheet. Please ask us if there is anything that is not clear to you or you would like more information about.

**What is the purpose of the study?**

Recent survey of the paediatric workforce participating in health research was published in October 2015. It showed that 81.6% of paediatric consultants do not have research as part of their programmed activities and 54.5% do not do any research at all.

The percentage of academic paediatric consultants has fallen from 12% to 3.8% between 1999 and 2013. In 2012, there were only about 200 paediatric professors, readers, and senior lecturers in the UK and the number of lecturers nationally, was 28, the lowest level ever. Over the next decade, 50% of current professors of paediatrics will retire, indicating the likelihood of further decline in leadership in children’s research.

The scant evidence-base for child healthcare is impeding the development of effective national guidelines and policy with less than 20% of outputs from the National Institute of Clinical Excellence applicable to children.

The RCPCH has commented that **Child health research is at ‘worryingly low’ level**.

Although, the RCPCH has done an extensive survey looking at the workforce involved in research, it was focused only at consultants and specialty doctors. We aim to collect information from paediatric trainees for barriers and challenges in pursuing involvement in research and academic roles.

**What will happen to me if I take part?**

You will be contacted to arrange a suitable time and place to carry out an interview. We envisage that the majority of these interviews will be conducted in person. If you have any questions about this study you can contact Khurram Mustafa (0113 206 7157) [khurram.mustafa@nhs.net](mailto:khurram.mustafa@nhs.net) to discuss this before you agree to take part.

**Do I have to take part?**

Participation is entirely voluntary. You may refuse to take part and you do not have to tell us why you do not want to take part. If you decide to take part you can still withdraw at any time without giving a reason.

**Are there any risks involved?**

There are no risks to taking part in the interview. You will not be asked to provide any patient specific information. This interview is entirely based on your understanding and experience of research and the associated challenges.

**What will happen to the data collected as part of the interview study?**

We aim to help identify the barriers faced and perceived by paediatric trainees that have led to the decline in research involvement. For this purpose, we will be taking one to one interviews with trainees at different levels to identify major themes. We have also developed an online that will be sent to all paediatric trainees in Yorkshire and the Humber.

In the end, we will organize a panel of experts to discuss the findings of this study. You will also be invited to that discussion. Although, you can opt out from this and we will not send you an invitation. You can decide not to attend this session at any point and you do not require to give us a reason for that.

As a result of discussion with paediatric trainees and experts in paediatric research, we hope to be able to suggest recommendations and action plans to help improve the current situation.

We would be happy to supply you with a copy of the results on request.

**Who is funding the study?**

My current post as a clinical leadership fellow is funded by Health Education England and I am based at Leeds Teaching Hospitals.

Thank you for taking the time to read this information sheet. If you know of any other paediatric trainee in Yorkshire and the Humber who might be interested to take part in this study, could you kindly suggest them to contact Khurram Mustafa on 0113 206 7157 or email khurram.mustafa@nhs.net
